# The Effect of the Physical Presence of Co-Players on Perceived Ostracism and Event-Related Brain Potentials in the Cyberball Paradigm

**DOI:** 10.1371/journal.pone.0071928

**Published:** 2013-08-09

**Authors:** Sarah Weschke, Michael Niedeggen

**Affiliations:** Department of Education and Psychology, Freie Universität Berlin, Berlin, Germany; University of Vienna, Austria

## Abstract

The affective and cognitive mechanisms elicited by the experience of social exclusion—or ostracism—have recently been explored using behavioral and neurocognitive methods. Most of the studies took advantage of the Cyberball paradigm, a virtual ball tossing game with presumed co-players connected via the internet. Consistent behavioral findings indicate that exclusion obviously threatens fundamental social needs (belonging, self-esteem, meaningful existence, and control) and lowers mood. In this study, we followed the question whether the credibility of the setting affects the processing of social exclusion. In contrast to a control group (standard Cyberball setup), co-players were physically present in an experimental group. Although the credibility of the virtual ball tossing game was significantly enhanced in the experimental group, self-reported negative mood and need threat were not enhanced compared to the control group. Event-related brain potentials (ERPs), however, indicated a differential processing of social exclusion. The N2 amplitude triggered by occasional ball receptions was significantly reduced in the experimental group. This effect was restricted for an early time range (130–210 ms), and did not extend to the following P3 components. The ERP effect in the N2 time range can be related to a differential social reward processing in ostracism if co-players are physically present. The lack of a corresponding correlate in the behavioral data indicates that some facets of ostracism processing are not covered by questionnaire data.

## Introduction

Ostracism is defined as “ignoring and excluding individuals or groups by individual or groups” [1]. As a framework to explicate the assumed reflexive and reflective reactions following ostracism, the need-threat model was proposed, stating that four fundamental needs, namely belonging, self-esteem, meaningful existence, and control, are threatened following ostracism [2]. Since belonging to a group is essential for physical and psychological health [3] the painful threat of the fundamental needs is assumed to serve as an early detection system to enhance motivation to reconstitute affiliation to other persons [4]. The mechanisms of perceiving and processing social exclusion have not only been studied using behavioral [1], but also neuroimaging [5], [6] and electrophysiological methods [7], [8], [9]. Both approaches were helpful in localizing the neuronal and cognitive networks involved.

Most of the behavioral and psychophysiological studies took advantage of the Cyberball paradigm [10], [11]. Here, participants are told to play a virtual ball tossing game with two – or more – other participants connected via the internet to measure visual imagination capabilities. In fact, the “co-players” are computer-generated and the probability of receiving the ball is experimentally manipulated, i.e. in the exclusion condition the participant hardly ever receives the ball. Despite its artificial character, several studies confirmed that fundamental social needs can be reliably threatened with the Cyberball game [12], [13].

Nevertheless, it can be questioned whether the effect of ostracism is independent from the credibility of the paradigm. Although there is evidence that ostracism can even be elicited when the participants knew that they were playing with computer-generated co-players [14], other studies have shown that the conviction of interacting with a computer or another human leads to huge differences in emotions and behavior in tasks [15], [16], [17]. For example, subjects interacting with a computer described them as behaving according to a design, whereas human beings are perceived as intentionally and rationally acting agents [18]. Moreover, the emotional state was found to be modulated in social exchange paradigms when human players – but not computers – were involved [19].

The studies aforementioned lead to the question of whether an increase of authenticity will change the ostracism effect induced in the Cyberball game. One possible approach is the introduction of co-players who are physically present. The presence of co-players might not only affect the credibility of the experimental situation, but also have an effect on the affective or cognitive processing of social exclusion. It is well known that behavior or perceptional decisions are influenced by the presence of other human beings [20], [21], even if they did not directly observe the behavior of the subject [22]. Also, automatic attitudes and spontaneous affective responses can be changed by the presence of other human beings [23], [24].

A further question is whether the effect of introducing co-players physically present can be measured by means of a retrospective method. In most of the experimental studies based on the Cyberball paradigm the Need Threat Questionnaire (NTQ) [10] was applied. The NTQ measures the effects of inclusion or exclusion on the perceived level of social need threat (belonging, self-esteem, meaningful existence, and control) and on negative mood. However, the NTQ – like other questionnaires – is applied subsequently to the experience of exclusion induced in the Cyberball game. In this respect, it seems advantageous to adopt an online measurement such as the recording of electrophysiological activity *during* the Cyberball game. Event-related potentials (ERPs) meet the requirements of investigating a pre-cognitive early detection system through its high temporal resolution. Previous studies have shown that ERPs time-locked to the event of not receiving the ball evoke a late prefrontal positivity interpreted as a coping mechanism as a reaction to exclusion, and enhanced N2 and P3 amplitudes [7], [9], [25].

Since the Cyberball paradigm shares some characteristics of the well-established Oddball paradigm [26], we focused our analysis on the critical event “ball possession” in a previous study [8]. The corresponding ERP probes are related to the subjective stimulus relevance, probability, and expectancy [27], [28], [29]. The results confirmed that an N2/P3 complex is (a) triggered when the player receives the ball, and (b) significantly enhanced when comparing exclusion (two co-players: 16% ball possession) with inclusion (two co-players: 33% ball possession). Finally, we observed significant correlations between the P3 complex, negative mood and perceived ostracism intensity. The latter confirms our assumption that components in the P3 complex can serve as a valid “online” indicator for ostracism expectancy [8].

These previous results triggered the experimental question whether retrospective reports (NTQ) and/or ERP correlates will be affected if the credibility of the Cyberball game is enhanced. For this reason, we compared the standard design (virtual presence of co-players) with a modified setup (physical presence of two – assumed – co-players). Since there is evidence that the source of exclusion does only have minimal influences on the NTQ data [14], we did not expect that the retrospectively reported experience of ostracism will be affected by the physical presence of co-players. We rather expected an effect on negative mood since a higher credibility of the social setting was found to modulate the affective state [19]. With respect to the ERP components, we assumed that ball possession will trigger a N2/P3 complex, and that specific components will reflect the involvement in the game and the presence of co-players: In the oddball-like setup [8], the N2 amplitude was determined by ball reception irrespective of the social interaction context (inclusion vs. exclusion). Since the component apparently indicates task relevance [30], we did not expect a modulation of this component. The following P3a, however, was enhanced in the exclusion block [8], and appears to reflect the activation of a conflict-based neural alarm system related to activity in the anterior cingulate cortex [31], [32]. Since its amplitude was also related to the affective state of the participant [8], we assumed that the P3a can be enhanced by the physical presence of co-players. As for the P3b, a corresponding modulation in amplitude was also observed in the exclusion condition [8]. Comparable to the oddball-design, the increase in amplitude as well as its relation to perceived ostracism was related to a modulation in subjective probability [29]. Since the component is therefore rather related to cognitive processes, such as memory updating or stimulus classification [33], we did not expect a modulation in our experimental setup.

## Materials and Methods

### Participants

The procedure was approved by the local ethics committee at the FU Berlin. Thirty-six healthy subjects participated in the experiment. Due to a high number of artifacts in the EEG, six participants had to be excluded, leaving 30 for analyses. The participants had self-reportedly no history of psychiatric or neurological disorders and were not taking medication affecting the central nervous system. They were recruited in the university environment and gave their written consent for participating. The subjects were randomly assigned to one of the experimental groups (Internet *n* = 15, 7 female, mean age  = 24.7 years; Physical Presence *n* = 15, 10 female, mean age  = 22.5 years). Since a cover story was required to induce the experimental effect, participants were informed about the experimental technique and aiming of the study afterwards. Participants got credit points for their studies.

### Task and Design

E-Prime 2 (Psychology Software Tools, Inc.) was used to present standardized instructions, the Cyberball game and to trigger EEG recording. All participants were told that they took part in a study testing visual imagination capabilities. To keep up this cover story, all participants first completed a short questionnaire (Vividness of Visual Imagery Questionnaire) about visual imagination ability [34].

**Figure 1:**
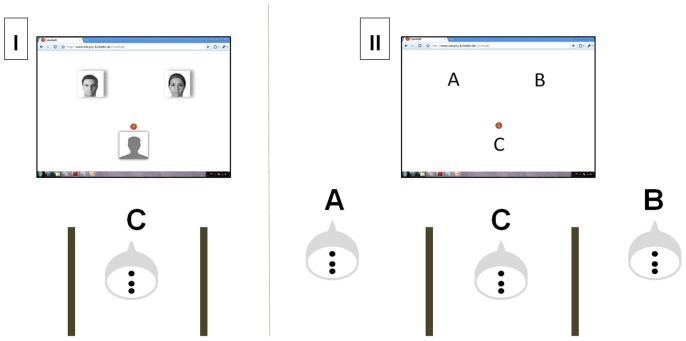
Experimental setting for the *Internet* (I) and *Presence* (II) condition. The real participant was always sitting at position C. In the *Internet* group, the two “co-players” were depicted by two photographs. In the *Presence* group, the confederates “A” and “B” pretended to be involved in the ball tossing game, which was actually possible only in 15 training trials at the beginning of the game. Please note that the photographs of co-players depicted do not refer to real persons, but are morphs of different portraits.

Setup for the group *Internet* followed the established Cyberball design [8]: Participants were told that they would play a ball tossing game with two other co-players connected via internet. In contrast, participants assigned to the group *Presence* ought to believe they were playing with the two other co-players present in the same room, who were actually confederates of the experimenter (see Figure 1). To enhance plausibility, the confederates also had electrodes fixed on their scalps. They greeted each other, but were told not to talk or interact in another way during the experiment. Earlier, the confederates were requested not to react on possible comments of the participant during the game.

Following the instructions and a short training introduction, all participants went through two blocks of the Cyberball game. Each block consisted of 200 ball throws and lasted about 7 minutes. In the block *Inclusion*, the participant received the ball in about one third of all ball throws (33 percent); in the following block *Exclusion*, the probability of getting the ball was marked down to 17 percent. The partial exclusion was necessary in order to record the ERP correlate of the event “ball possession”, and we have shown previously that partial exclusion is also sufficient to induce a significant effect of ostracism [8].

After the exclusion block, two NTQ questionnaires were handed out. The subjects were told to retrospectively fill out the questionnaires, the first one regarding the first block, and the second one regarding the second block. To make the separation of the two experimental blocks less difficult, one part of the ball tossing game had to be imagined in the meadow and the other game on a beach (the order was counterbalanced across subjects). As already indicated in our previous study [8], the NTQ can reliably differentiate between the *Inclusion* and *Exclusion* condition, even if ratings on the first block are to be delivered with a temporal delay.

Participants were also asked to rate if they believed that their co-players were computer-generated. After completing all questionnaires, the subjects were informed about the real aim of the study. In the group *Presence* it was made sure that participants were informed about the scripted behavior of the two co-players.

### EEG Recording and data analysis

#### EEG data

EEG data were recorded from 3 active electrode positions (Fz, Cz, Pz). Previous experiments had shown that these positions are highly sensitive to record the components of interest [8]. Ag/AgCl skin electrodes were fixed on the scalp with EC2 Electrode Cream (Grass Technologies). Active electrodes (impedance <10 kΩ) were referenced to linked earlobes (< 5 kΩ), with AFz serving as ground. Vertical and horizontal electrooculogram (EOG) were also recorded to control for ocular artefacts (< 20 kΩ). Biosignals were recorded continuously with EEG BioAmplifiers and Psylab recording software (Contact Precision Instruments, London), then analyzed with BrainVision Analyzer 2 (Brain Products GmbH, Germany). Offline, data were band-pass filtered (0.3 to 30 Hz) and notch filtered (50 Hz). EEG segments were created (–100 to 800 ms after the participant received the ball) according to the condition *Inclusion* or *Exclusion* and baseline-corrected (–100 to 0 ms). Subsequently, a semiautomatic artifact rejection was performed, eliminating segments containing eye blinks, muscular artifacts or high alpha activity. Since there were more segments for ball possession in the condition *Inclusion* by definition, the number of EEG segments was randomly chosen to adjust it to the number of segments obtained in the condition *Exclusion*. Averages and grand averages were calculated, separately for the two experimental groups, conditions and three electrode positions. Grand averages revealed distinctive components in three consecutive time ranges: 130 to 210 ms (N2), 240 to 300 ms (P3a), and 300 to 410 ms (P3b). Mean amplitudes in these time windows were exported and analyzed using SPSS (version 19, IBM). Repeated measures ANOVAs were calculated including the between-subject factor “group assignment” (*Internet* vs. *Presence*) and the within-subject factors “condition” (*Inclusion* vs. *Exclusion*) and “electrode position” (Fz vs. Cz vs. Pz). Degrees of freedom and *p*-values were corrected according to Greenhouse-Geisser, if indicated, and corrected *p*-values will be reported in the following.

#### Behavioral data

For each participant, data of the NTQ and additional questions were read in SPSS (version 19, IBM) and NTQ scales belonging, self-esteem, meaningful existence and control, and an additional scale included in the NTQ measuring negative mood were computed (all items were rated on a 1 to 5 Likert scale, with NTQ scales having a potential range between 1 and 5 and negative mood between 4 and 20). The data were analyzed running a repeated measures ANOVA including the between-subject factor “group” (*Internet* vs. *Presence*) and the within-subject factor “condition” (*Inclusion* vs. *Exclusion*). To assess the conviction regarding the cover story that the subjects were playing with other human beings the participants finally rated the statement “the co-players were computer-generated” on a 1 to 5 Likert scale after completing the NTQ (see Table 1), also analyzed by a repeated measures ANOVA including the between-subject factor “group” and the within-subject factor “condition” and one-way comparisons within each condition.

**Table 1:** Behavioral data

	Internet		Presence	
NTQ Scale	Inclusion	Exclusion	Inclusion	Exclusion
belonging	3.8000 (0.5746)	2.5333 (0.9241)	4.1556 (0.6769)	2.6667 (1.1055)
self esteem	3.6000 (0.6068)	3.1778 (0.5019)	3.4667 (0.6016)	3.2222 (0.7834)
meaningful existence	4.2667 (0.8281)	3.6000 (1.0925)	4.3111 (1.0348)	3.2000 (1.1464)
control	2.3111 (0.6954)	1.6667 (0.7346)	2.0667 (0.7684)	1.4000 (0.4216)
negative mood	8.5333 (2.5317)	12.6000 (2.7464)	8.5000 (2.9641)	11.1333 (3.4355)
Estimated percentage ball possession	30.5333 (10.3776)	12.9333 (5.4703)	28.2000 (6.6030)	15.2667 (7.5448)
Item “Co-players were computer-generated.”	3.7333 (1.1629)	4.0667 (1.0328)	1.5333 (0.8338)	2.4000 (1.2984)

Behavioral results of the *Internet* and *Presence* group for the *Inclusion* and *Exclusion* blocks are depicted. Mean values and standard deviations (in brackets) are presented.

## Results

### Behavioral Data

Behavioral data (see Table 1) showed that the presence of two supposed co-players led to a reduced acceptance of the item “co-players were computer-generated” compared to the group *Internet*. This was confirmed by a main effect of the factor “group” in an ANOVA, *F*(1,28)  = 31.608, *p*<.001, η^2^ = .530. In addition, a main effect of the factor “condition”, *F*(1,28)  = 8.624, *p* = .007, η^2^ = .235, showed the assumption to interact with a computer was increased during exclusion. This effect was not modulated by group membership (interaction *F*(1,28)  = 1.703, *p* = .202). In one-way comparisons for *Inclusion* and *Exclusion*, respectively, the group difference was also confirmed (*Internet* vs. *Presence*: Inclusion: *F*(1,28)  = 35.456, *p*<.001, η^2^ = .559; Exclusion: *F*(1,28)  = 15.138, *p* = .001, η^2^ = .351).

In each NTQ scale, the expected decrease in the condition *Exclusion* as compared to *Inclusion* regarding the four fundamental needs was obtained, as well as a decrease in the estimation of ball possession (see Table 1). The analysis of the NTQ scales confirmed the expected significant reduction for the scales “belonging”, *F*(1,28)  = 35.162, *p*<.001, η^2^ = .557, “self esteem”, *F*(1,28)  = 7.377, *p* = .011, η^2^ = .209, “meaningful existence”, *F*(1,28)  = 12.782, *p* = .001, η^2^ = .313, and “control”, *F*(1,28)  = 12.782, *p*<.001, η^2^ = .378. Moreover, the data indicated a significant increase in “negative mood”, *F*(1,28)  = 28.595, *p*<.001, η^2^ = .505. There was neither a main effect of group assignment (*p*-value ≥.215 for each of the four NTQ scales and negative mood) nor an interaction of the factors “condition” and “group” (*p*-value ≥.180 for each NTQ scale and negative mood) obtained.

In addition, estimation of ball possession by the participants (see Table 1) differed significantly between *Inclusion* and *Exclusion*, *F*(1,28)  = 68.875, *p*<.001, η^2^ = .711, independently of group membership (main effect group: *F*(1,28)  = 1.609, *p* = .215; interaction: *F*(1,28) <1).

### ERP Data

**Figure 2:**
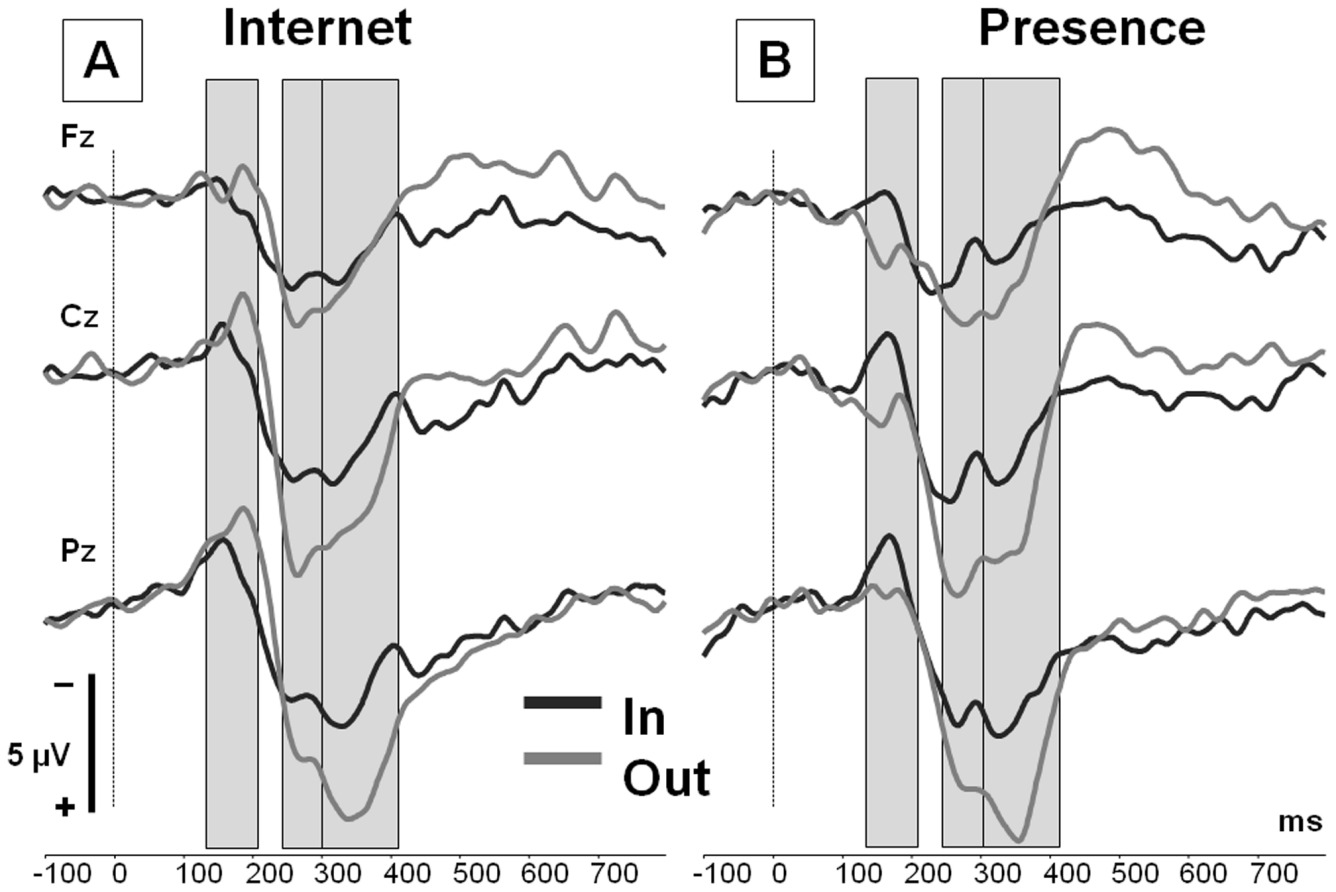
ERP data. Grand-averaged ERPs for the event “ball possession of the participant” in the *Inclusion* (dark grey) and *Exclusion* condition (light grey) recorded from the electrode positions Fz, Cz and Pz. Three time windows are highlighted: 130–210 ms (N2), 240–300 ms (P3a), and 300–410 ms (P3b). (A) Superimposition of the ERP traces in the group *Internet*: Co-players are assumed to be connected via internet. (B) Superimposition of the ERP traces in the group *Presence*: Co-players are physically present in the lab.

The grand-averaged ERPs evoked by the event “ball possession” are depicted in Figure 2. Three time ranges were exported for further analyses: The N2 range (130 to 210 ms) was marked by a negative peak at about 180 ms, the P3a (240 to 300 ms) by a fronto-central positivity at about 260 ms, and the P3b (300 to 410 ms) by a late parietal positivity peaking at about 350 ms. The mean amplitudes and standard deviations for the three components are presented in Table 2.

**Table 2:** Electrophysiological data

		Internet		Presence	
ERPcomponent	Electrode position	Inclusion	Exclusion	Inclusion	Exclusion
N2	Fz	0.0994 (2.1858)	–0.5316 (3.2778)	0.6305 (1.8439)	2.1040 (2.7702)
	Cz	–0.8220 (2.5238)	–1.8387 (3.7477)	–0.3012 (2.3122)	1.6751 (3.5746)
	Pz	–1.5536 (2.4044)	–2.9526 (3.4894)	–1.5166 (2.1295)	–0.3623 (3.7714)
P3a	Fz	3.1338 (3.2622)	4.3682 (5.0819)	2.6029 (3.1952)	4.5014 (3.6478)
	Cz	3.8761 (4.6757)	6.9462 (5.5924)	4.1932 (4.1449)	7.9977 (2.5751)
	Pz	3.5246 (4.0801)	5.3526 (4.2705)	4.0239 (3.2034)	6.2860 (3.0721)
P3b	Fz	2.2193 (3.2225)	2.5030 (2.8470)	1.5775 (2.8913)	2.7261 (3.7795)
	Cz	2.8520 (3.9656)	5.2044 (2.3687)	2.9440 (2.8710)	5.5544 (3.2789)
	Pz	3.3914 (3.1557)	7.1318 (3.2258)	3.9171 (3.3838)	7.3046 (2.9446)

Mean values of ERP components of the *Internet* and *Presence* group for the *Inclusion* and *Exclusion* blocks are depicted. ERPs were recorded from Fz, Cz and Pz. Mean values and standard deviations (in brackets) in microvolt are presented for three distinct time frames (N2: 130–210 ms, P3a: 240–300 ms, P3b: 300–410 ms).

#### N2 (130–210 ms)

In both groups, the N2 component was clearly visible in the conditions *Inclusion* and *Exclusion*, mostly pronounced at parietal leads (main effect of electrode position, *F*(2,56)  = 29.373, *p*<.001, η^2^ = .512). As depicted in Figure 2, the component was reduced in the group *Presence* during exclusion, whereas a contrary effect was observed in the group *Internet*. This was confirmed by an interaction of the factors “group” and “condition”, *F*(1,28)  = 6.648, *p* = .015, η^2^ = .192. Post-hoc comparison within each group confirmed a significant reduction of the component in the group *Presence*, *F*(1,14)  = 7.031, *p* = .019, η^2^ = .334, most pronounced at fronto-central leads. In contrast, no significant modulation in the group *Internet*, *F*(1,14)  = 1.603, *p* = .226, was found. The effect cannot be attributed to inherent differences between groups during inclusion, since the corresponding post-hoc comparisons did not indicate any differences between groups in the condition *Inclusion* (*p*-value ≥.477 for each electrode).

#### P3a (240–300ms)

Receiving the ball evoked an early positivity most prominent at Cz (main effect of electrode: *F*(2,56)  = 11.498, *p*<.001, η^2^ = .291) and more pronounced in the exclusion condition (main effect of condition: *F*(1,28)  = 10.197, *p* = .003, η^2^ = .267). The effect of condition was not modulated by the factor “group assignment”, *F*(1,28) <1, but it was affected by electrode position, *F*(2,56)  = 5.689, *p* = .012, η^2^ = .169. Post-hoc tests for the electrode positions separately revealed that the effect of condition is restricted to central, *F*(1,28)  = 15.277, *p* = .001, η^2^ = .353, and parietal, *F*(1,28)  = 7.233, *p* = .012, η^2^ = .205, electrode leads.

#### P3b (300–410 ms)

A P3b succeeded the P3a, mainly pronounced at the parietal electrode position (main effect of electrode: *F*(2,56)  = 40.542, *p*<.001, η^2^ = .591). Furthermore, it was more pronounced in *Exclusion*, *F*(1,28)  = 24.991, *p*<.001, η^2^ = .472. This effect of condition was modulated by electrode position, *F*(2,56)  = 15.764, *p*<.001, η^2^ = .360, but not by group assignment (*F*(1,28) <1). The corresponding post-hoc tests revealed effects of condition at central, F(1,28)  = 18.601, p<.001, η^2^ = .399, and parietal, F(1,28)  = 46.259, p<.001, η^2^ = .623, electrode positions. Group assignment did not modulate the effects.

## Discussion

### Summary of results

The presence of co-players had a significant effect on the credibility of the cover story: According to the questionnaire, subjects assigned to the group *Presence* were more convinced that they were playing with human beings. This effect held for both conditions, *Inclusion* and *Exclusion*. Nevertheless, the higher credibility did not affect NTQ ratings: In both groups, the decrease in the need threat scale scores (belonging, self-esteem, meaningful existence and control) and increase in negative mood when participants were excluded was comparable and no overall group difference was obtained.

ERP data revealed a group difference in the early time range: In the temporal range of the N2 component (130–210 ms), a significant reduction was obtained only in the group *Presence* when participants were excluded. In the P3 range (P3a: 240–300 ms, P3b: 300–410), social exclusion led to a significant increase in amplitudes. This effect, however, was not modulated by the presence of co-players.

### Effect of credibility on the NTQ

Although credibility was increased, perceived ostracism – as measured with the NTQ – was not affected by the physical presence of co-players. This is in line with previous studies showing that participants even felt excluded when they knew that their co-players were computer-generated [14]. The findings also confirmed that there are no differences between social- and cyber-ostracism in the condition *Exclusion -* at least for the needs “belonging” and “meaningful existence” [35]. However, the aforementioned study also found that the communication medium obviously affected other needs, namely “control” and “self-esteem”. This differential effect regarding the four fundamental needs can be explained by the experimental setup used. Williams et al. [35] compared computer-mediated communication (i.e. communication in a chat room) with face-to-face discussions, and both variants describe a more-realistic scenario than the virtual ball-tossing game.

Beside the four fundamental needs aforementioned, we did not observe a differential effect on negative mood which would have been in line with previous results comparing interactions with humans or computers [19]. However, a previous Cyberball study [13] indicated a unidirectional relationship between negative mood and need threat as induced by exclusion: an increase in need threat triggers an increase in negative mood, but not *vice versa*. It is therefore unlikely that the presence of co-players selectively affects negative mood, but not need threat in social exclusion.

Further, our data showed no effect of the physical presence of social interaction partners within the Cyberball paradigm. At first sight, this supports the notion that NTQ data are primarily related to the assumed early pre-attentive processes triggered by exclusion in the Cyberball paradigm, and that this mechanism is not modulated by the physical presence of co-players or other manipulations in the game and does not necessarily require intention [12], [13], [14].

### ERP: Effects in the N2 range

Within the time range of the N2 component (130–210 ms), we obtained an effect of credibility. This effect was limited to *Exclusion* and can therefore not be explained by the physical of (assumed) co-players presence *per se*. The modulation of the amplitude is either related to a decrease in amplitude of the N2 component, or to the superimposition of a unique early ERP positivity.

As stated above, we assumed the N2 to be elicited in the case of ball possession in both conditions, *Inclusion* and *Exclusion,* independently of group assignment [8]. Following our hypothesis, the N2 amplitude reflects the degree of task relevance [30] since ball possession demands a motor reaction from the participant. The differential modulation within this temporal range, however, indicated an early effect of the presence of co-players on social exclusion. Three possible accounts will be discussed in turn.

#### Modulation of a conflict-based neural alarm system

In a recent ERP study using the Cyberball paradigm, an enhancement of N2 amplitude was assumed to signal the activation of a neural alarm system when excluded [9]. Its activation is triggered by a pre-conscious conflict-monitoring system [36]. Following this idea, the decrease of the N2 amplitude observed in our data would signal a down-regulation of the alarm system in the group *Presence*. However, Figure 2 indicates that the N2 was already expressed in *Inclusion*, and was therefore not specifically related to partial exclusion in the ball tossing game [8]. Moreover, the latency of the N2 effect observed (200–320 ms, [9]) rather refers to the P3a time range analyzed in our study. We will therefore consider the activation of this system in the following section.

#### Modulation of a defense sensitivity system

Within an emotional oddball design, a reduction of N2 amplitude was triggered by pleasant stimuli, and assumed to reflect a reduction in the defense system sensitivity [37]. A corresponding process might be elicited in our group *Presence*: Here, the reception of the ball in the exclusion condition signaled a re-involvement in the game and thereby reduces social threat. According to previous results, however, we have to take into account that a N2 reduction in the group *Presence* is expected to be associated with a corresponding modulation of the P3 amplitudes [37]. As stated in the result section, this was not the case in our study.

#### Modulation of the social reward signal

The visual inspection of the grand-averaged ERPs also indicates a transient positivity at frontal and central leads in the group *Presence* within the N2 time range (see Figure 2.B). Therefore, we also have to consider the superimposition of a P2-like process in the exclusion condition. A corresponding ERP process has been reported in reward processing [38], [39]: Here, a fronto-central positivity was elicited after receiving unexpected rewards, and neutralized the N200 component. In our study, receiving the ball was comparable to a social reward, and it was probably more valuable in the (partial) exclusion condition. Following this idea, ball possession was treated as a social reward only if the credibility of an interaction with human beings was given. This hypothesis is substantiated by a neuroimaging study indicating that face-to-face interactions activate the reward system to a greater extent than recorded social interactions [40].

In sum, the ERP effect in the N2 time range indicates that the presence of co-players affects an early processing stage involved in the appraisal of social exclusion. We assume that the occasional involvement in the game within an *Exclusion* rally serves as a social reward signal if co-players are present. It is important to note, however, that this process is not directly reflected by the N2, but rather by an independent ERP positivity (P2) superimposed. The more direct account – N2 amplitude as an indicator for social threat – appears to be less convincing since the P3 amplitude is not modulated as well [37], [41].

### ERP: Effects in the P3 range

ERP data confirmed results that the P3a and P3b components are sensitive to ostracism manipulation in the Cyberball paradigm [8]. The P3a and P3b effect was also in line with the participants’ estimation of ball possession (see Table 1): Both experimental groups provided a valid estimation when comparing the change from inclusion to exclusion, and in both groups a comparable increase of P3 components was observed. Since the effects in the N2 time range (see above) have been related to a differential reward processing between the groups *Internet* and *Presence*, a corresponding effect might be also expected for the P3 amplitudes [42], [43]. However, P3 amplitudes were obviously less sensitive to the magnitude of a “social” reward provided by co-players physically present.

As for the P3a, our previous study [8] provided evidence that it was related to the activation of an early alarm system – already implemented in the seminal model on social exclusion [2]. This system was supposed to determine the affective response to exclusion and to be located in the anterior cingulate cortex [5], [44]. A previous ERP study also postulated the activation of a conflict-based alarm system in a corresponding temporal window (200–300 ms, [9]), but it was related to a conflict N2 [36]. Within this theoretical framework, one might conclude that the alarm system is activated independently of the credibility of the experimental setup. Accordingly, we did not observe an effect on the negative mood of our participants.

As for the P3b, we previously related its amplitude to the subjective expectancy of social exclusion, i.e. occasional ball reception within an exclusionary rally does not meet the participants’ expectation of continuous exclusion [8]. As suggested for the parietal, oddball-triggered P3 complex [26], [44], the component is related to controlled processing, such as memory- or context-updating operations and the expectancy towards feedback [45]. The impact of subjective expectancies on ostracism intensity has already been highlighted in studies on rejection sensitivity in healthy and clinical samples [46], [47].

We propose that the P3 amplitudes and the NTQ data – replicating earlier results [8] – rely on a common stage in the cognitive processing of social exclusion, namely the expectancy of receiving the ball in the exclusion condition. Both, retrospective behavioral measures and P3 effects were primarily determined by the subjective probability to get involved in the ball tossing game. The credibility of the Cyberball game – significantly increased by the presence of co-players – does obviously not affect the psychological processes subserving the participants’ expectancies on involvement.

## Conclusion

The results indicate that psychophysiological (ERP) data reflect different experiences of social exclusion related to facets not covered by the items of the applied self-report measure (NTQ). One facet might be the enhancement of the perception of social rewards when playing with human beings. ERP data are therefore more capable to detect transient processes within the Cyberball game. To cover differential aspects in the processing of social exclusion, the recording of an online measurement is beneficial. When applying the Cyberball game, imaging or electrophysiological techniques as well as analyses of facial expression [5], [7], [9], [48] will provide further insight into the dynamics of processing which may remain hidden in a mere retrospective design.

## References

[1] Williams KD (2007) Ostracism. Annual Review of Psychology 58: 425–452. 10.1146/annurev.psych.58.110405.085641 16968209

[2] Williams KD, Zadro L (2001) Ostracism: On being ignored, excluded and rejected. In: Leary MR, editor. Interpersonal rejection. New York: Oxford University Press. pp. 21–53.

[3] Baumeister RF, Leary MR (1995) The Need to Belong - Desire for Interpersonal Attachments as a Fundamental Human-Motivation. Psychological Bulletin 117: 497–529. 7777651

[4] Williams KD, Zadro L (2005) Ostracism: the indiscriminate early detection system. In: Williams KD, Forgas JP, Hippel WV, editors. The social outcast - ostracism, social exclusion, rejection, and bullying. New York: Psychology Press. pp. 19–34.

[5] Eisenberger NI, Lieberman MD, Williams KD (2003) Does rejection hurt? An fMRI study of social exclusion. Science 302: 290–292. 14551436 10.1126/science.1089134

[6] Eisenberger NI (2012) The neural bases of social pain: evidence for shared representations with physical pain. Psychosom Med 74: 126–135. 22286852 10.1097/PSY.0b013e3182464dd1 PMC3273616

[7] Crowley MJ, Wu J, McCarty ER, David DH, Bailey CA, et al (2009) Exclusion and micro-rejection: event-related potential response predicts mitigated distress. Neuroreport 20: 1518–1522. 19829163 10.1097/WNR.0b013e328330377a PMC4457507

[8] Gutz L, Kupper C, Renneberg B, Niedeggen M (2011) Processing social participation: an event-related brain potential study. Neuroreport 22: 453–458. 21558970 10.1097/WNR.0b013e3283476b67

[9] Themanson JR, Khatcherian SM, Ball AB, Rosen PJ (2012) An event-related examination of neural activity during social interactions. Soc Cogn Affect Neurosci. 10.1093/scan/nss058 PMC3739919 22577169

[10] Williams KD, Cheung CKT, Choi W (2000) Cyberostracism: Effects of being ignored over the Internet. Journal of Personality and Social Psychology 79: 748–762. 11079239 10.1037//0022-3514.79.5.748

[11] Williams KD, Jarvis B (2006) Cyberball: A program for use in research on interpersonal ostracism and acceptance. Behavior Research Methods 38: 174–180. 16817529 10.3758/bf03192765

[12] Gonsalkorale K, Williams KD (2007) The KKK won't let me play: Ostracism even by a despised outgroup hurts. European Journal of Social Psychology 37: 1176–1186.

[13] van Beest I, Williams KD (2006) When inclusion costs and ostracism pays, ostracism still hurts. Journal of Personality and Social Psychology 91: 918–928. 17059310 10.1037/0022-3514.91.5.918

[14] Zadro L, Williams KD, Richardson R (2004) How low can you go? Ostracism by a computer is sufficient to lower self-reported levels of belonging, control, self-esteem, and meaningful existence. Journal of Experimental Social Psychology 40: 560–567.

[15] Sundar SS, Nass C (2000) Source orientation in human-computer interaction - Programmer, networker, or independent social actor? Communication Research 27: 683–703.

[16] Kiesler S, Sproull L, Waters K (1996) A prisoner's dilemma experiment on cooperation with people and human-like computers. Journal of Personality and Social Psychology 70: 47–65. 8558408 10.1037//0022-3514.70.1.47

[17] Miwa K, Terai H (2012) Impact of two types of partner, perceived or actual, in human-human and human-agent interaction. Computers in Human Behavior 28: 1286–1297.

[18] Gallagher HL, Jack AI, Roepstorff A, Frith CD (2002) Imaging the intentional stance in a competitive game. Neuroimage 16: 814–821. 12169265 10.1006/nimg.2002.1117

[19] van't Wout M, Kahn RS, Sanfey AG, Aleman A (2006) Affective state and decision-making in the Ultimatum Game. Experimental Brain Research 169: 564–568. 16489438 10.1007/s00221-006-0346-5

[20] Zajonc RB (1965) Social Facilitation. Science 149: 269–274. 14300526 10.1126/science.149.3681.269

[21] Asch SE (1956) Studies of Independence and Conformity.1. A Minority of One against a Unanimous Majority. Psychological Monographs 70: 1–70.

[22] Rittle RH, Bernard N (1977) Enhancement of Response Rate by Mere Physical Presence of Experimenter. Personality and Social Psychology Bulletin 3: 127–130.

[23] Sinclair S, Lowery BS, Hardin CD, Colangelo A (2005) Social tuning of automatic racial attitudes: The role of affiliative motivation. Journal of Personality and Social Psychology 89: 583–592. 16287420 10.1037/0022-3514.89.4.583

[24] Castelli L, Tomelleri S (2008) Contextual effects on prejudiced attitudes: When the presence of others leads to more egalitarian responses. Journal of Experimental Social Psychology 44: 679–686.

[25] Crowley MJ, Wu J, Molfese PJ, Mayes LC (2010) Social exclusion in middle childhood: rejection events, slow-wave neural activity, and ostracism distress. Soc Neurosci 5: 483–495. 20628967 10.1080/17470919.2010.500169 PMC2991408

[26] Squires NK, Squires KC, Hillyard SA (1975) 2 Varieties of Long-Latency Positive Waves Evoked by Unpredictable Auditory-Stimuli in Man. Electroencephalography and Clinical Neurophysiology 38: 387–401. 46819 10.1016/0013-4694(75)90263-1

[27] Johnson R Jr, Donchin E (1980) P300 and stimulus categorization: two plus one is not so different from one plus one. Psychophysiology 17: 167–178. 7375618 10.1111/j.1469-8986.1980.tb00131.x

[28] Potts GF, Patel SH, Azzam PN (2004) Impact of instructed relevance on the visual ERP. International Journal of Psychophysiology 52: 197–209. 15050377 10.1016/j.ijpsycho.2003.10.005

[29] Duncan-Johnson CC, Donchin E (1977) On Quantifying Surprise - The Variation of Event-Related Potentials with Subjective-Probability. Psychophysiology 14: 456–467. 905483 10.1111/j.1469-8986.1977.tb01312.x

[30] Wijers AA, Okita T, Mulder G, Mulder LJ, Lorist MM, et al (1987) Visual search and spatial attention: ERPs in focussed and divided attention conditions. Biological Psychology 25: 33–60. 3447636 10.1016/0301-0511(87)90066-4

[31] Polich J (2007) Updating p300: An integrative theory of P3a and P3b. Clinical Neurophysiology 118: 2128–2148. 17573239 10.1016/j.clinph.2007.04.019 PMC2715154

[32] Volpe U, Mucci A, Bucci P, Merlotti E, Galderisi S, et al (2007) The cortical generators of P3a and P3b: a LORETA study. Brain Res Bull 73: 220–230. 17562387 10.1016/j.brainresbull.2007.03.003

[33] Polich J, Kok A (1995) Cognitive and biological determinants of P300: an integrative review. Biological Psychology 41: 103–146. 8534788 10.1016/0301-0511(95)05130-9

[34] Marks DF (1973) Visual Imagery Differences in Recall of Pictures. British Journal of Psychology 64: 17–24. 4742442 10.1111/j.2044-8295.1973.tb01322.x

[35] Williams KD, Govan CL, Croker V, Tynan D, Cruickshank M, et al (2002) Investigations into differences between social- and cyberostracism. Group Dynamics-Theory Research and Practice 6: 65–77.

[36] Yeung N, Botvinick MM, Cohen JD (2004) The neural basis of error detection: conflict monitoring and the error-related negativity. Psychol Rev 111: 931–959. 15482068 10.1037/0033-295x.111.4.939

[37] Mardaga S, Hansenne M (2009) Personality modulation of P300 wave recorded within an emotional oddball protocol. Neurophysiol Clin 39: 41–48. 19268846 10.1016/j.neucli.2008.12.005

[38] Holroyd CB, Krigolson OE, Lee S (2011) Reward positivity elicited by predictive cues. Neuroreport 22: 249–252. 21386699 10.1097/WNR.0b013e328345441d

[39] Potts GF, Martin LE, Burton P, Montague PR (2006) When things are better or worse than expected: The medial frontal cortex and the allocation of processing resources. Journal of Cognitive Neuroscience 18: 1112–1119. 16839285 10.1162/jocn.2006.18.7.1112

[40] Redcay E, Dodell-Feder D, Pearrow MJ, Mavros PL, Kleiner M, et al (2010) Live face-to-face interaction during fMRI: A new tool for social cognitive neuroscience. Neuroimage 50: 1639–1647. 20096792 10.1016/j.neuroimage.2010.01.052 PMC2849986

[41] Stanford MS, Vasterling JJ, Mathias CW, Constans JI, Houston RJ (2001) Impact of threat relevance on P3 event-related potentials in combat-related post-traumatic stress disorder. Psychiatry Res 102: 125–137. 11408052 10.1016/s0165-1781(01)00236-0

[42] Yeung N, Sanfey AG (2004) Independent coding of reward magnitude and valence in the human brain. J Neurosci 24: 6258–6264. 15254080 10.1523/JNEUROSCI.4537-03.2004 PMC6729539

[43] Wu Y, Zhou X (2009) The P300 and reward valence, magnitude, and expectancy in outcome evaluation. Brain Res 1286: 114–122. 19539614 10.1016/j.brainres.2009.06.032

[44] Polich J, Criado JR (2006) Neuropsychology and neuropharmacology of P3a and P3b. International Journal of Psychophysiology 60: 172–185. 16510201 10.1016/j.ijpsycho.2005.12.012

[45] Hajcak G, Holroyd CB, Moser JS, Simons RF (2005) Brain potentials associated with expected and unexpected good and bad outcomes. Psychophysiology 42: 161–170. 15787853 10.1111/j.1469-8986.2005.00278.x

[46] Romero-Canyas R, Downey G, Berenson K, Ayduk O, Kang NJ (2010) Rejection sensitivity and the rejection-hostility link in romantic relationships. J Pers 78: 119–148. 20433615 10.1111/j.1467-6494.2009.00611.x

[47] Renneberg B, Herm K, Hahn A, Staebler K, Lammers CH, et al (2012) Perception of social participation in borderline personality disorder. Clin Psychol Psychother 19: 473–480. 22076727 10.1002/cpp.772

[48] Staebler K, Renneberg B, Stopsack M, Fiedler P, Weiler M, et al (2011) Facial emotional expression in reaction to social exclusion in borderline personality disorder. Psychol Med 41: 1929–1938. 21306661 10.1017/S0033291711000080

